# Supplementary material for: T-Cell Receptor Repertoire Sequencing and Its Applications: Focus on Infectious Diseases and Cancer
Source: Int J Mol Sci. 2022 Aug 2;23(15):8590. doi: 10.3390/ijms23158590 (PMC9369427; doi:10.3390/ijms23158590)
Supplement: Supplementary file 1 [file ijms-23-08590-s001.zip › ijms-1832455-supplementary.pdf]

Supplementary Table S1. TCR sequencing kits and services overview.

Company	Service/ kit	Kit name	Starting material	Input (depends on source)	Library prep method	Total library prep time	Hands-on-time for library prep	Chains	CDR regions	Sequencing platform	Data analysis
**BGI**	service	/	RNA	525ng - 3,5ug	Mplex-PCR	/	/	TCRα TCRβ	CDR3	Illumina: HiSeq	40 working days from sample QC acceptance to filtered raw data availability
					5'-RACE	/	/	TCRα TCRβ	CDR1 CDR2 CDR3	Illumina: MiSeq	
**Adaptive Biotechnologies**	service/ kit	ImmunoSEQ Human T-cell Receptor Beta (hsTCRB)	gDNA cDNA	400ng - 10ug	Mplex-PCR:	8-10 hours	4-5 hours	TCRβ	CDR3	Illumina: HiSeq, MiSeq	ImmunoSEQ-Analyzer
	service	/	gDNA cDNA	/	Mplex-PCR	/	/	TCRα TCRδ TCRγ	CDR3		
**iRepertoire, Inc.**	kit	Bulk Primer	gDNA RNA	100ng	amplicon rescued Mplex-PCR	6-8 hours	20 min-3 hours	TCRα TCRβ	CDR3	Illumina: HiSeq, MiSeq, Novaseq.	iRweb
		iR-Profile	RNA		amplicon rescued Mplex-PCR	/	/	TCRα TCRβ TCRδ TCRγ	CDR2 CDR3		
		iR-Flex Automation	RNA		amplicon rescued Mplex-PCR or dimer avoided Mplex-PCR	/	/	TCRα TCRβ TCRδ TCRγ	/		
	service	RepSeq	RNA	75ng - 200ng	amplicon rescued Mplex-PCR	/	/	TCRα TCRβ TCRδ TCRγ	CDR3		
		RepSeq+	RNA		dimer-avoided multiplex PCR	/	/		/		
**Clontech TaKaRa Bio USA, Inc.**	kit	SMARTer Human TCR a/b Profiling Kit v2 (TCRv2)	RNA	1ng - 1µg	SMART (5'-RACE)	6,5-7,5 hours	2-3 hours	TCRα TCRβ	CDR1 CDR2 CDR3	Illumina: HiSeq, MiSeq	Cogent NGS Immune Profiler Software

[Illumina]()	kit	AmpliSeq for Illumina TCR beta-SR Panel	gDNA RNA	1ng	Mplex-PCR	5,5-7,5 hours	1,5 hours	TCRβ	CDR3	Illumina: MiniSeq, MiSeq, NextSeq	MiXCR Immune Repertoire Analyzer
		AmpliSeq for Illumina Immune Repertoire Plus, TCR beta Panel	RNA	10ng - 1ug				TCRβ	CDR1 CDR2 CDR3		
[Thermo scientific]()	kit	Oncomine™ TCR Beta-LR Assay	RNA	10ng - 1ug	Mplex-PCR	48 hr sample-to-results time		TCRβ	CDR1 CDR2 CDR3	Ion GeneStudio S5	Ion Reporter Software v5.6
		Oncomine™ TCR Beta-SR Assay	gDNA RNA	50ng - 1ug				TCRβ	CDR3		Ion Reporter Software v5.10
[Invivoscribe Technologies, Inc.]()	kit	LymphoTrack TRB Assay	gDNA	50ng minimum	Mplex-PCR	/	/	TCRβ	/	Illumina: MiSeq	LymphoTrack MiSeq Software, Lymphotrack MRD Software
		LymphoTrack TRG Assay			Mplex-PCR	/	/	TCRγ	/		
	service	TRB Clonality Assay	gDNA	500ng	PCR	/	/	TCRβ	/	/	12 to 14 working days
[ArcherDX, Inc.]()	kit	Immunoverse TCR Beta/Gamma	RNA	25ng-2ug	Mplex-PCR (unidirectional gene-specific primers)	8-5 hours	<3,5 hours	TCRβ TCRδ	/	Illumina: MiSeq, NextSeq	RNA Immune Repertoire pipeline (Archer Analysis)
		Immunoverse TCR Alpha/Delta						TCRα TCRγ	/		
[Creative Biolabs]()	service	Magic™ TCR Repertoire Sequencing Service	RNA	/	Magic™ IR-seq platform	/	/	TCRα TCRβ TCRδ TCRγ	CDR3	/	/
[MiLaboratories]()	kit	Human TCR	RNA	/	UMI 5'-RACE	/	/	TCRα TCRβ	CDR3	Illumina: MiSeq, NextSeq	MiXCR Platform
		Human Multiplex TCR	RNA	/	Mplex-PCR	/	/	TCRα TCRβ	/	Illumina: MiSeq, NextSeq, HiSeq, Novaseq	
		Human DNA TCR	DNA	/	Mplex-DNA	/	/	TCRα TCRβ	/		
	service	5'RACE WITH UMI	RNA	high quantity	UMI 5'-RACE	/	/	TCRα TCRβ TCRδ	/	/	MiXCR Platform. Turnaround

								TCRγ			time is routinely about 1-3 months
		RNA mutiplex	RNA	low quantity	Mplex-PCR	/	/	TCRα TCRβ	CDR3		
		DNA multiplex	DNA	/	Mplex-PCR	/	/				
